# Effects of Dominant Associated Bacteria *Agrobacterium radiobacter* Bx.F4 and *Delftia tsuruhatensis* Bx.Q2 on the Physiological Traits of *Bursaphelenchus xylophilus*: Insights from RNA-Seq Analysis

**DOI:** 10.3390/microorganisms14010010

**Published:** 2025-12-19

**Authors:** Mengyu Chen, Chenglei Qin, Yujiang Sun, Qunqun Guo, Congbei Lv, Han Wang, Zijin Zhou, Guicai Du, Ronggui Li

**Affiliations:** 1 College of Life Sciences, Qingdao University, Qingdao 266071, China; cmy2017@hotmail.com (M.C.); qinchenglei@qdu.edu.cn (C.Q.); gqunqun@163.com (Q.G.);; 2 Qingdao Landscape and Forestry Integrated Service Center, Qingdao 266061, China; qdsfmai@163.com

**Keywords:** PWN-associated bacteria, population growth, lifespan, feeding ability, pathogenicity, transcriptome

## Abstract

To investigate the role of bacteria associated with pine wood nematodes (PWNs), *Bursaphelenchus xylophilus* (Steiner & Buhrer) Nickle, we isolated two dominant bacterial strains (*Agrobacterium radiobacter* Bx.F4 and *Delftia tsuruhatensis* Bx.Q2) from *B. xylophilus* with different pathogenicity (highly pathogenic F and weakly pathogenic Q) identified in previous studies. In this study, *Agrobacterium radiobacter* Bx.F4 and *Delftia tsuruhatensis* Bx.Q2 were inoculated, respectively, to aseptic PWNs to identify the effects of dominant nematode-associated bacteria on the motility, egg laying, population growth, lifespan, feeding ability, and pathogenicity of PWNs. The results showed that *Agrobacterium radiobacter* Bx.F4 could significantly enhance the motility, egg-laying, population growth, lifespan, feeding ability, and pathogenicity of PWNs, while *Delftia tsuruhatensis* Bx.Q2 exhibited the opposite effects. Furthermore, using RNA-sequencing analysis, we found that the longevity regulatory pathways, along with the JNK, Wnt, and FOXO signaling pathways related to these genes were clearly enriched. These results indicate that dominant bacterial strains associated with *B. xylophilus* may regulate various metabolic activities within the nematode, thereby exerting a significant influence on its physiological state and pathogenicity. This also provides a potential strategy for the biocontrol of pine wilt disease (PWD).

## 1. Introduction

The pine wood nematode (*Bursaphelenchus xylophilus* [Steiner & Buhrer] Nickle, PWN) represents a highly damaging plant-parasitic nematode that is the causal agent of pine wood nematode disease (PWD), or pine wilt disease [1]. With high reproductive rates, destructive capacity, and environmental adaptability, PWN rapidly causes pine wilt, resulting in significant ecological and economic damage to forest ecosystems [2].

PWN, originating in North America, was first identified in the United States in 1929 but caused no significant ecological damage locally. By the 1970s, transnational timber trade facilitated its cross-border transmission via wooden packaging materials, leading to its initial establishment in Japan. Its subsequent rapid spread across Asia affected China and South Korea [3,4]. Since the 21st century, this pathogen has invaded Europe, with confirmed outbreaks in Portugal, Spain, and the United Kingdom [5]. Current global monitoring data indicate its presence in at least 18 countries across Asia, Europe, North America, and Africa [6]. Notably, Asia’s extensive contiguous pine forests have rendered it the region most severely impacted by pine wilt disease [7,8]. Globally, PWN-susceptible tree species include *Pinus massoniana*, *Pinus thunbergii*, *Pinus densiflora*, and other pine genus members, as well as non-pine conifers such as *Picea* spp. (spruce) and *Abies* spp. (fir) [4,9]. The cumulative area affected by PWD in China has reached millions of hectares, posing a formidable challenge in prevention and control [10]. The alarming spread rate and severity of PWD in China have inflicted disastrous impacts on forest resources, triggering severe ecological and economic consequences [11].

Research has revealed a complex mutualistic relationship between PWN and its associated bacteria, which enhances its pathogenicity and adaptability. Bacteria carried by PWN such as *Pseudomonas fluorescens* and *Pseudomonas putida* significantly increased nematode fecundity, reproduction rate, and adult growth, while the nematodes, in turn, promoted bacterial proliferation. This mutualistic symbiosis suggests long-term co-evolution, with metabolic interactions, enhancing both organisms’ adaptability [12]. PWNs and the associated fungus, *Sporothrix* sp. 1, exhibit a synergistic relationship, where their interaction enhances PWN pathogenicity and accelerates pine tree mortality. When PWN fed on *Sporothrix* sp. 1, lipid metabolism gene expression and metabolite production were upregulated, leading to increased palmitoleic acid levels through both uptake and de novo synthesis, thereby enhancing nematode fecundity. Conversely, increased nematode populations promote fungus dispersal [13]. Palmitoleic acid acts as a molecular link, enhancing the fitness and reproductive success of both species and providing insights for other investigations of biological co-evolution. The co-evolutionary relationship between bacteria and pine wood nematodes is a key factor affecting the occurrence and spread of pine wilt disease. There are significant differences in the types of endophytic bacteria associated with pine wood nematodes from different pine species, and the dynamic changes and diversity of bacterial communities have a significant impact on the pathogenicity of pine wood nematodes, especially during the host switching stage in the nematode life cycle [14]. This dynamic symbiotic relationship reflects the synergistic effect of bacteria and nematodes in adaptive evolution. Certain symbiotic bacteria are capable of modulating the pine tree’s antioxidant system, thereby inhibiting host defense responses [15]. The antioxidant metabolites they secrete, such as antioxidant enzymes and peptides, can lower the levels of reactive oxygen species (ROS) within the pine, consequently weakening the tree’s immune response and establishing a more suitable environment for pine wood nematode reproduction [16,17,18,19]. The expansion of gene families in the pine wood nematode genome related to the detoxification of terpenes (such as cytochrome P450 and short-chain dehydrogenase) may have partially benefited from the long-term selective pressure of bacterial metabolic products [20,21]. In addition, pine wood nematodes can produce cellulase (β-1,4-endoglucanase) and pectinase to digest plant tissues, which may be due to horizontal gene transfer from bacteria and fungi to the nematodes [22,23]. PWN-associated bacteria enhance nematode tolerance to terpenoids and phenolic compounds, facilitating survival and reproduction within pine trees that contain these defensive compounds [24,25]. The presence of bacteria also enhances PWN cold resistance, allowing them to maintain physiological activity and population stability under low-temperature conditions [26]. These adaptive enhancement mechanisms provide a crucial foundation for further investigations into the PWN-bacteria interaction, furthering our understanding of this symbiotic relationship’s role and significance within the ecosystem.

Given the critical role of bacteria in PWN’s adaptability, this study aims to investigate the relations between dominant bacterial communities and PWN strains of varying virulence, and analyze bacterial-mediated gene expression in PWN using transcriptomics. This research will advance our understanding of the drivers behind PWN virulence variation and elucidate the synergistic pathogenic mechanisms of nematode-bacteria interactions.

## 2. Materials and Methods

### 2.1. PWN and Nematodes Culturing

*Bursaphelenchus xylophilus* (Steiner & Buhrer) Nickle isolates F (highly virulent) and Q (weakly virulent), which were originally isolated from Fushan Forestry Park and Qingdao University campus, respectively, are characterized isolates preserved in our laboratory and were utilized as they have been in our prior research for their differing levels of virulence [27]. For subculture inoculation, the isolated nematodes were transferred to PDA plates fully colonized by *Botrytis cinerea* (pre-grown at 25 °C in the dark for 7 days), which served as a food source.

### 2.2. Isolation and Identification of Culturable Bacteria from Nematodes

Culturable bacteria were isolated from the bodies of PWNs by the reported method [28], and the strains were cultured in nutrient broth (NB) liquid medium at 25 °C for 24 h. Total genomic DNA was extracted from the bacteria of *B. xylophilus* using the E.Z.N.A.^®^ Bacterial DNA Kit (Omege Biotech, Norcross, GA, USA). The 16S rDNA was amplified by primers 27F (5′-AGAGTTTGATCCTGGCTCAG-3′) and 1492R (5′-TACCTTGTTACGACTT-3′). The PCR reaction was carried out according to the method of Yuan et al. [29], and the PCR product sequencing was completed by Sangon Biotech Co., Ltd. (Shanghai, China). The similarity of the 16S rDNA sequence was compared with the existing sequences available in the NCBI GenBank database using a BLAST (https://www.ncbi.nlm.nih.gov/tools/primer-blast/, accessed on 30 August 2024) search. The sequence alignment and the phylogenetic tree were constructed by MEGA11 software using the Neighbor-Joining method based on the Kimura 2-parameter (K2P) model [30]. Bootstrap analysis was performed with 1000 replicates.

### 2.3. Treatment of Nematodes and Bacteria

To prepare aseptic PWN, the nematodes were extracted via Bellman funnel overnight, washed thrice with sterilized distilled water, disinfected with 9% hydrogen peroxide for 50 min, and rinsed thoroughly. Then, they were exposed to a mixture of 4% ciprofloxacin and 4% ceftriaxone sodium for 8 h before being rinsed again. Finally, PWN culturing on nutrient agar (NA) plates at 25 °C for 48 h, a bacterial staining check, and PCR analysis of 16S rDNA were used to verify the absence of bacterial contamination.

To promote stable colonization of specific bacteria associated with *B. xylophilus*, the bacterial strains were first cultured in NB medium with shaking until the optical density (OD) at 600 nm reached approximately 0.8 (mid-log phase). The bacterial cells were collected by centrifugation and gently resuspended in sterile distilled water. Subsequently, the bacterial suspension was mixed with surface-sterilized and viability-confirmed nematodes and co-incubated statically at 25 °C for 12 h. After incubation, the nematodes were gently washed three times with sterile distilled water to thoroughly remove unbound free bacteria. Finally, the number of bacteria firmly attached to the nematode surface was quantified using the plate colony counting method [31].

In this study, *Agrobacterium radiobacter* Bx.F4, isolated from *B. xylophilus* isolates F (highly virulent), and *Delftia tsuruhatensis* Bx.Q2, isolated from *B. xylophilus* isolates Q (weakly virulent), were used as experimental materials. Both isolated bacterial strains were identified as belonging to the dominant genera associated with *B. xylophilus* based on comparative analysis with metagenomic data from prior studies [27]. Each bacterium was co-cultured separately with aseptic PWNs. The resulting nematode samples were designated as experimental groups and named S-Bx.F4 and S-Bx.Q2, respectively.

### 2.4. Egg-Laying Ability of Nematodes

Ten male and ten unmated female PWNs (all late J4 stage) from the aseptic PWN group (control group, CK), S-Bx.Q2, and S-Bx.F4 were randomly selected and transferred to 24-well tissue culture plates containing sterile distilled water and incubated at 25 °C. After allowing them to mate in the dark for 36 h, the number of eggs in each well was counted under a stereomicroscope (Motic SMZ-168, Fujian, China) at 80× magnification. CK, S-Bx.Q2, and S-Bx.F4 had six repeat treatments each. This data were recorded as the number of eggs laid per female nematode.

### 2.5. Measurement of Motility and Lifespan of Nematodes

The head thrashing frequency of *B. xylophilus* is a key indicator for evaluating its motility. Adult PWNs from the control group (CK), S-Bx.Q2, and S-Bx.F4 were randomly selected and transferred to 24-well tissue culture plates containing sterile distilled water for incubation at 25 °C. Within each well, 10 live adult nematodes were randomly chosen for observation, and their head-thrashing frequency per minute was recorded.

To calculate the survival rate of PWNs, the number of live nematodes in each well was counted daily until all nematodes were dead. The final recorded day was then taken as the lifespan of the nematodes. CK, S-Bx.Q2, and S-Bx.F4 had six repeat treatments each.

### 2.6. Analysis of Population Growth and Feeding Ability of Nematodes

Equal numbers of PWNs (approximately 100 per dish) from the control group (CK), S-Bx.Q2, and S-Bx.F4 were individually inoculated onto 6 cm Petri dishes overlaid with *B. cinerea* and cultured at 25 °C. Feeding zones were observed daily and quantified using ImageJ software version 1.8.0 (National Institutes of Health, Bethesda, MD, USA).

After the *B. cinerea* on the plates had been consumed by the nematodes, the nematodes were collected using the Baermann funnel method [32], and their numbers were recorded as an indicator of the population growth of the nematodes.

### 2.7. Pathogenicity Analysis of Nematodes

Equal numbers of PWNs (approximately 5000 per seedling) from the control group (CK), S-Bx.Q2, and S-Bx.F4 were inoculated into two-year-old *P. thunbergii* seedlings, respectively, with four replicates per treatment. The inoculation method was based on previously reported methods [33]. The disease severity index (DSI) reflected the difference in the wilting degree of indirect species among different treatments, and the disease incidence reflected the difference in wilting number among repeats of each group. The severity of infection in *P. thunbergii* was divided into five levels (Table 1). The disease grading standard and DSI were calculated according to Yu et al. [34].

(1) $$\mathrm{Disease}\;\mathrm{incidence}\;\left(\%\right)=\frac{\sum \mathrm{number}\;\mathrm{of}\;\mathrm{infected}\;\mathrm{plants}\;\mathrm{with}\;\mathrm{symptoms}}{\mathrm{Total}\;\mathrm{number}\;\mathrm{of}\;\mathrm{plants}}\;\times \;100\;$$

(2) $$\mathrm{Disease}\;\mathrm{severity}\;\mathrm{index}=\frac{\sum \mathrm{number}\;\mathrm{of}\;\mathrm{diseased}\;\mathrm{plant}\;\;\times \;\mathrm{disease}\;\mathrm{grad}}{\mathrm{Total}\;\mathrm{number}\;\mathrm{of}\;\mathrm{plants}\;\;\times \;\mathrm{highest}\;\mathrm{disease}\;\mathrm{grade}}\;\times \;100\;$$

### 2.8. RNA Isolation, cDNA Synthesis, and RNA Sequence

Total RNA was extracted from approximately 20,000 aseptic nematodes (control group, CK), S-Bx.Q2, and S-Bx.F4, respectively, using TRIzol^®^ reagent (Invitrogen, Carlsbad, CA, USA) and following the manufacturer’s protocol. RNA quality was evaluated by electrophoresis on RNase-free agarose gels and analyzed using an Agilent 2100 Bioanalyzer (Agilent Technologies, Santa Clara, CA, USA). RNA concentration was determined using both a NanoPhotometer spectrophotometer (Thermo Fisher Scientific, Waltham, MA, USA) and a Qubit 2.0 fluorometer (Thermo Fisher Scientific, Waltham, MA, USA). Complementary DNA libraries were prepared with the TruSeqTM RNA Sample Prep Kit (Illumina, Inc., San Diego, CA, USA), size-selected using Certified Low Range Ultra Agarose (Bio-Rad, Hercules, CA, USA), and quantified with high sensitivity using TBS380 Picogreen (Invitrogen, USA). Final sequencing was conducted on an Illumina HiSeq4000 platform by Biozeron Biotechnology Co., Ltd. (Shanghai, China).

### 2.9. Quantitative Real-Time PCR

Total RNA was extracted using the TRIzol^®^ reagent, and cDNA was synthesized using the PrimeScript RT Reagent Kit (Takara Bio Inc., Kusatsu, Japan). cDNA was precisely quantified using a NanoPhotometer spectrophotometer. The qPCR was then performed on ABI7000 Fast (Thermo Fisher Scientific, USA) using the Talent qPCR PreMix (SYBR Green) Kit (TIANGEN, Beijing, China), following the manufacturer’s protocol. Primer sequences are listed in Table 2. The actin gene (GenBank accession number: EU100952) was selected as the reference gene for qPCR. The relative expression levels of the CK group were set as 1 (*n* = 6). The experiment was performed twice, with three replications for each treatment, and gene expression levels were quantified using the 2^−∆∆Ct^ method.

### 2.10. Sequencing Data Analysis

Trimmomatic (http://www.usadellab.org/cms/index.php?page=trimmomatic, accessed on 1 September 2024) was used to preprocess raw sequencing data for high-quality reads.

The specific parameters were as follows: adapter removal; 3′ end trimming (Q < 20); reads with >10% Ns removed; discard reads <75 bp after trimming. Goatools (https://github.com/tanghaibao/GOatools, accessed on 5 September 2024) and KOBAS (http://bioinfo.org/kobas, accessed on 10 September 2024) were used for differential gene expression analysis (Fisher’s exact test; FDR < 0.05, absolute fold change ≥ 2).

RNA sequencing data have been deposited in the China National Center for Bioinformation (CNCB) database, GSA, and are accessible under accession number CRA024370 (https://ngdc.cncb.ac.cn/gsa/search?searchTerm=CRA024370, accessed on 3 April 2025).

### 2.11. Statistical Analysis

All biological experiments were conducted in triplicate, with six technical replicates for each treatment, unless stated otherwise. Data are expressed as mean ± standard deviation (SD). All parameters were calculated using SPSS Statistics 17.0 (IBM, Armonk, NY, USA). The statistical analysis was performed with One-Way Analysis of Variance (* *p* < 0.05, ** *p* < 0.01, *** *p* < 0.001) in GraphPad Prism 10 (GraphPad Software, San Diego, CA, USA).

## 3. Results

### 3.1. Culturable Bacteria Associated with PWNs and Recolonization of PWNs

**Figure 1:**
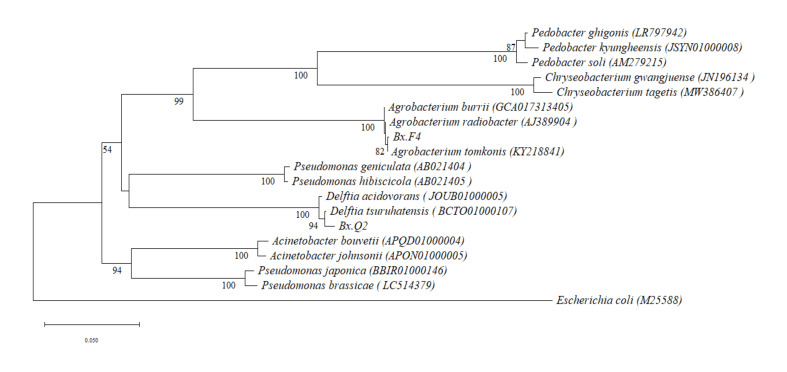
Phylogenetic trees of bacteria isolated from *Bursaphelenchus xylophilus* based on 16S rDNA. Support values of bootstrap > 50% are indicated at nodes.

Two dominant bacterial strains were isolated from PWN isolate F and isolate Q, designated Bx.F4 and Bx.Q2, respectively. Phylogenetic analysis based on 16S rDNA sequences revealed that strain Bx.F4 showed 99.71% similarity to *Agrobacterium radiobacter*, while strain Bx.Q2 exhibited 99.23% with *Delftia tsuruhatensis* (Figure 1). These findings aligned with our previous metagenomic studies of PWN-associated bacteria [27].

Aseptic PWNs were prepared, and their sterile effect was confirmed by incubating PWN on NA plates, performing a bacterial staining check, and by PCR analysis of 16S rDNA (Figures S1 and S2). A plate-counting assay indicated that *A. radiobacter* Bx.F4 and *D. tsuruhatensis* Bx.Q2 were successfully recolonized on aseptic PWNs, with an average bacteria count of 218 CFU and 199 CFU per nematode for triple repetition, respectively.

### 3.2. Analysis of Egg-Laying Ability of Different PWNs

**Figure 2:**
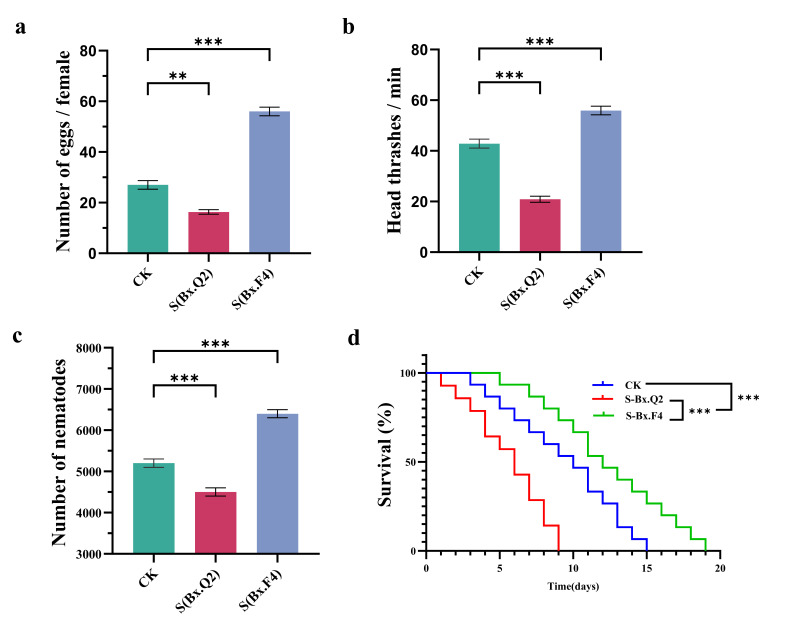
Analysis of two bacterial treatments on key physiological parameters of the pine wood nematode (PWN). (**a**) Effect of egg-laying on PWN (*n* = 6). (**b**) Effect of head thrashing on PWN (*n* = 6). (**c**) Effect of PWN population growth (*n* = 6). (**d**) Effect of PWN survival rate (*n* = 6). Data are expressed as mean ± standard deviation (SD). The statistical significance was performed with One-Way Analysis of Variance (** *p* < 0.01, *** *p* < 0.001).

The statistical analysis of PWN egg-laying ability demonstrated that in the aseptic PWNs group (control group, CK), each female nematode produced 31.0 ± 1.8 eggs. For PWNs associated with Bx.Q2 (S-Bx.Q2) and Bx.F4 (S-Bx.F4), the number of eggs laid per female nematode was 20.0 ± 1.3 and 60.0 ± 2.1, respectively. Specifically, egg production in S-Bx.Q2 showed a reduction of approximately 35.48%, while it increased by approximately 93.54% for S-Bx.F4 (Figure 2a), which certified that bacterial strains associated with PWN significantly influenced the egg-laying ability of nematodes.

### 3.3. Motility, Population Growth, and Survival Rate of Different PWNs

The statistical analysis of motility assessment revealed that in the aseptic PWNs group (CK), the head thrashing frequency per minute was 40 ± 1.58. In S-Bx.Q2 and S-Bx.F4 groups, the head thrashing frequency per minute were 23.0 ± 0.8 and 59.0 ± 0.7, respectively. When compared with the control group, there were significant differences in both the S-Bx.Q2 and S-Bx.F4 groups (*p* < 0.001). To be specific, the head thrashing frequency diminished by roughly 42.5% in S-Bx.Q2 and rose by about 47.5% in S-Bx.F4 (Figure 2b).

After the *B. cinerea* on the plates had been consumed by the nematodes (approximately 7 days), the results of population growth analysis revealed significant differences among nematode groups. The aseptic PWNs group (CK) produced 5398 ± 132 nematodes, and the S-Bx.Q2 group showed reduced reproduction (4113 ± 121 nematodes), representing a 23.8% decrease compared to CK (*p* < 0.001). Conversely, the S-Bx.F4 group exhibited enhanced population growth (6517 ± 109 nematodes), corresponding to a 20.73% increase relative to CK (*p* < 0.001) (Figure 2c).

Nematode survival analysis revealed distinct mortality patterns across experimental groups. The S-Bx.Q2 group exhibited complete mortality by 9 d, while the CK group reached 100% mortality at 15 d. Notably, the S-Bx.F4 treated nematodes demonstrated extended survival until 19 d. These results demonstrated strain-specific biological effects: S-Bx.Q2 showed accelerated lethality compared to controls, whereas S-Bx.F4 appeared to prolong nematode survival. The 10-day difference between S-Bx.Q2 and S-Bx.F4 mortality endpoints is shown in Figure 2d.

### 3.4. Feeding Ability of Different PWNs

**Figure 3:**
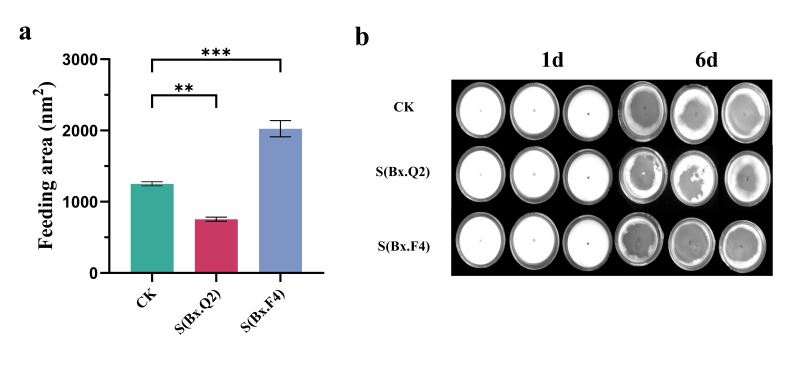
Analysis of two bacterial treatments on feeding zones of the pine wood nematode (PWN). (**a**) Effect of feeding area on PWN (*n* = 6). (**b**) Effect of feeding area on PWN in plate (*n* = 6). Data are expressed as mean ± standard deviation (SD). The statistical analysis was performed with One-Way Analysis of Variance (** *p* < 0.01, *** *p* < 0.001).

Feeding zone analysis revealed significant differences among experimental groups. The aseptic PWNs group (CK) displayed a baseline feeding area of 1252.25 ± 49.31 mm^2^ six days after inoculation. In contrast, the S-Bx.Q2 group showed markedly reduced feeding activity (753.82 ± 49.88 mm^2^), representing a 39.8% decrease compared to CK (*p* < 0.01). Conversely, the S-Bx.F4 group exhibited enhanced feeding behavior with an area of 2024.21 ± 61.66 mm^2^, corresponding to a 61.64% increase relative to controls (*p* < 0.001) (Figure 3a,b).

### 3.5. Pathogenicity Analysis of Different PWNs

**Figure 4:**
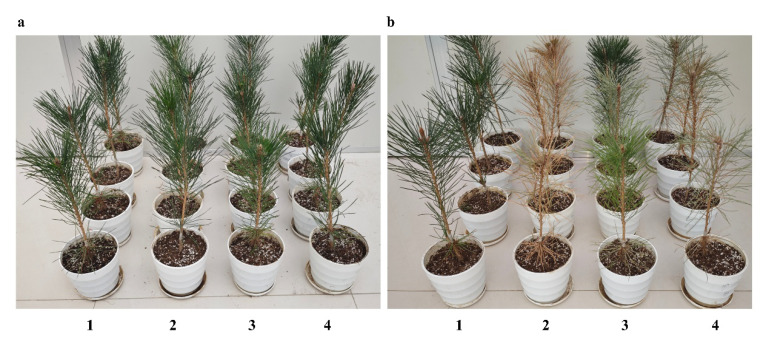
Inoculation of two-year-old *P. thunbergii* seedlings with different pine wood nematodes. (**a**) 1 dpi. (**b**) 60 dpi. 1. Sterilized water. 2. S-Bx.F4. 3. Aseptic PWNs. 4. S-Bx.Q2.

Pathogenicity assays were conducted by inoculating two-year-old *P. thunbergii* seedlings with sterile water, aseptic PWNs (CK), S-Bx.Q2 (aseptic PWNs carrying the *D. tsuruhatensis* Bx.Q2), and S-Bx.F4 (aseptic PWNs carrying the *A. radiobacter* Bx.F4), respectively (Figure 4a).

At 60 days post-inoculation, the health status of the seedlings differed markedly across treatment groups (Figure 4b). Seedlings inoculated with sterile water remained healthy, with a disease severity index (DSI) of 0. Those inoculated with aseptic PWNs (CK) showed slight wilting, corresponding to a DSI of 12.5. Seedlings treated with the S-Bx.Q2 bacterial strain exhibited moderate wilting and a DSI of 50. In contrast, all seedlings inoculated with the S-Bx.F4 bacterial strain had wilted and died, resulting in a DSI of 100 (Table 3).

### 3.6. Characteristics of the Transcriptomes of PWNs

Transcriptome sequencing of aseptic PWN (CK), S-Bx.Q2, and S-Bx.F4 generated 398,182,724 reads, of which 387,979,812 were clean (97.44%) and were retained after fastp filtering. Trinity de novo assembly produced 23,322 unigenes with an average length of 1283 bp (Table 4). All samples exhibited Q20 exceeding 98.75% and Q30 surpassing 96.53%, with a GC content of 45–46.28% (Table 5). Thus, the sample quality was sufficient for follow-up analysis and study. Subsequent functional annotation showed that 17,833 unigenes (76.5%) matched to the NCBI NR database, while 5014 (21.5%) and 9116 (39.1%) were annotated in the GO and SWISS databases, respectively (Table 6).

Gene Ontology (GO) analysis systematically categorized gene functions into three fundamental domains: Biological Process (BP), Cellular Component (CC), and Molecular Function (MF).

**Figure 5:**
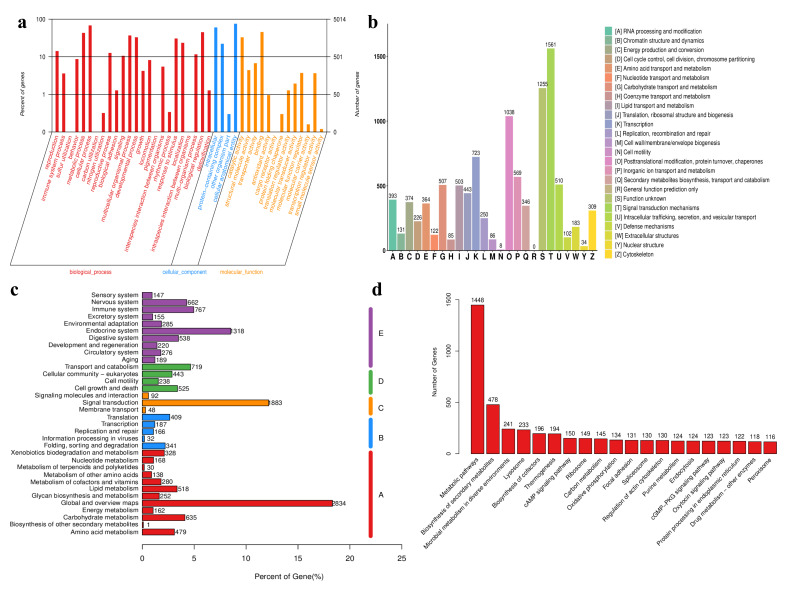
Functional annotation and pathway enrichment analysis of unigenes. (**a**) Gene Ontology (GO) annotation of unigenes. (**b**) Cluster of Orthologous Groups of proteins (COG) function classification of Unigenes. (**c**) Kyoto Encyclopedia of Genes and Genomes (KEGG) annotation of unigenes. A: cellular processes; B: environmental information processing; C: genetic information processing; D: metabolism; E: organismal systems. (**d**) Top 20 KEGG pathways of unigenes.

In the BP category, “cellular process” (GO:0009987) emerged as the most prevalent term (68.12%), followed by “biological regulation” (GO:0044699; 45.05%), “metabolic process” (GO:0008152; 43.05%), and “multicellular organismal process” (GO:0032501; 36.87%). Additional significant BP annotations included developmental processes (32.92%), stimulus response (30.61%), cellular localization (23.45%), and reproduction (14.16%). Lower frequency but biologically relevant terms encompassed signaling (10.52%), behavior (8.67%), locomotion (8.17%), and growth (4.2%). CC annotations revealed “cellular anatomical entity” (GO:0110165) as the predominant classification (75.34%), with “intracellular” components (GO:0005622) representing 60.37% of annotations. Protein complexes (GO:0032991) accounted for 22.17% of CC terms. The MF domain was characterized by a high representation of binding activities (GO:0005488; 45.51%) and catalytic functions (GO:0003824; 32.98%), with additional annotations for transporter activity (GO:0005215) and structural molecule activity (GO:0005198) (Figure 5a).

KOG functional classification successfully annotated 11,027 unigenes (47.3% of total 23,322). Excluding the “S” category (function unknown), the predominant functional classifications were distributed as follows: signal transduction mechanisms (“T”) represented the largest group with 1561 unigenes, followed by secondary metabolite biosynthesis (“O”; 1038) and transcription (“K”; 723) (Figure 5b).

KEGG pathway analysis successfully mapped 6052 unigenes (25.9% of total 23,322) to 252 distinct metabolic pathways. The annotated genes were distributed across five major functional categories: Cellular Processes (5825 genes; predominant category), Organismal Systems (4557 genes), Genetic Information Processing (2023 genes), Metabolism (1925 genes), and Environmental Information Processing (1135 genes). (Figure 5c).

KEGG pathway analysis revealed the top 20 most enriched pathways, with “metabolic pathways” showing the highest representation (1448 unigenes). Subsequent highly represented pathways included: “biosynthesis of secondary metabolites” (478 unigenes), “microbial metabolism in diverse environments” (241 unigenes), “lysosome” (233 unigenes), “biosynthesis of cofactors” (196 unigenes), and “thermogenesis” (194 unigenes) (Figure 5d).

### 3.7. Differential Gene Expression Analysis Between CK and S-Bx.F4

**Figure 6:**
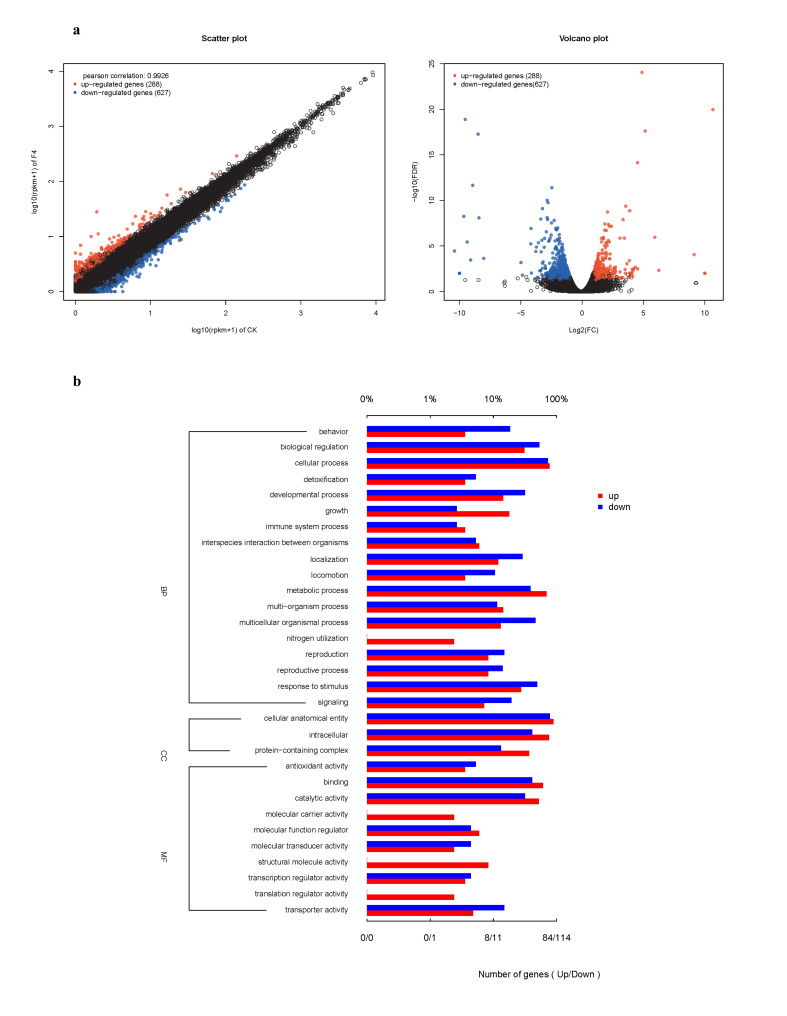
Functional annotation and enrichment analysis of DEGs between CK and S-Bx.F4. (**a**) EDGs visualized by Scatter Plot and Volcano Plot. (**b**) Gene Ontology (GO) analysis of the upregulated and downregulated genes.

Comparative analysis of the differentially expressed genes (DEGs) between CK and S-Bx.F4 identified a total of 915 DEGs, including 288 upregulated genes and 627 downregulated genes (Figure 6a).

GO analysis revealed that upregulated DEGs primarily participated in cellular (65) and metabolic (58) processes, followed by biological regulation (26) and response to stimulus (23), while downregulated DEGs were enriched in cellular (83), biological regulation (61), multicellular organismal (53), and developmental (36) processes within the Biological Process category. For Cellular Component (CC), upregulated DEGs mainly localized to cellular anatomical entities (75) and intracellular components (64), whereas downregulated DEGs showed similar patterns (89 and 47, respectively). Molecular Function (MF) analysis indicated binding (51 upregulated, 47 downregulated) and catalytic activity (44 upregulated, 36 downregulated) as predominant functions (Figure 6b).

**Figure 7:**
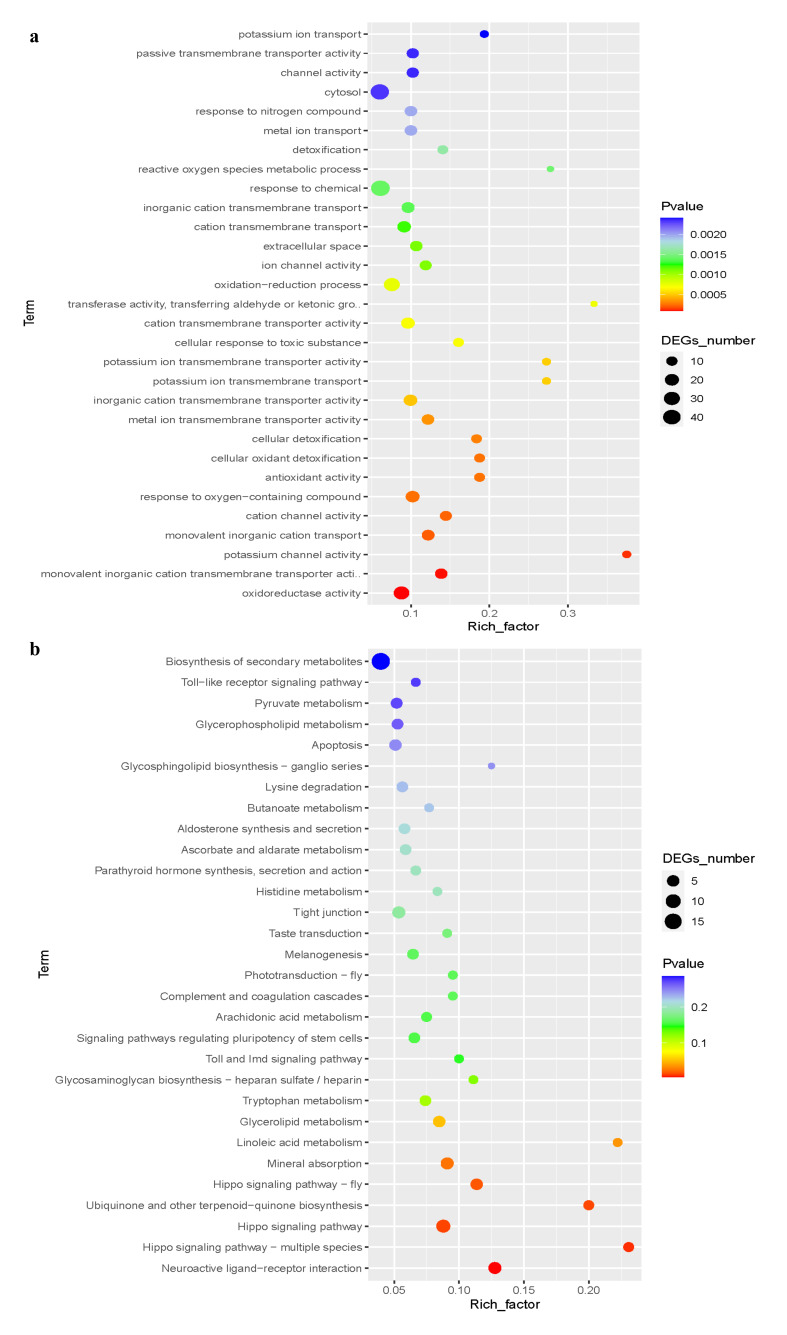
Functional annotation and enrichment analysis of DEGs between CK and S-Bx.F4. (**a**) EDGs visualized by Scatter Plot and Volcano Plot. (**b**) Gene Ontology (GO) analysis of the upregulated and downregulated genes.

GO enrichment analysis identified key biological processes affected in treatment group S-Bx.F4 compared to control CK. The most significantly altered processes included cytosol organization (46 DEGs: 37 upregulated, 9 downregulated) and chemical response (46 DEGs: 37 upregulated, 9 downregulated). The oxidation-reduction pathway showed substantial changes (48 DEGs: 18 upregulated, 35 downregulated), along with oxidoreductase activity (28 DEGs: 18 upregulated, 10 downregulated). Notably, passive transmembrane transport (13 downregulated DEGs), channel activity (13 downregulated DEGs), and metal ion transport (14 DEGs: 1 upregulated, 13 downregulated) were predominantly downregulated (Figure 7a).

KEGG enrichment analysis of differentially expressed genes (DEGs) between treatment group S-Bx.F4 and control group CK revealed significant enrichment in the top 30 metabolic pathways. The “Biosynthesis of secondary metabolites” pathway was the most significant pathway and is usually related to plant adaptation to environmental stress or the accumulation of secondary metabolites. The enrichment of the “Toll-like receptor signaling pathway” suggested active immune-related responses, possibly reflecting the changes in defense mechanisms against pathogens. The enrichment of pathways such as “Pyruvate metabolism” and “Glycerophospholipid metabolism” revealed key changes in energy metabolism and lipid metabolism processes, which might be related to cellular energy supply or the adjustment of membrane structures (Figure 7b).

Transcriptomic differential expression analysis revealed significant upregulation of several key genes. As shown in Table 7, these included astacin protease inhibitor, cytochrome c oxidase subunit II, ribosyldihydronicotinamide dehydrogenase (NQO2), zona pellucida 2-like, zona pellucida 4-like, extracellular superoxide dismutase, zona pellucida 3-like, MKK7, axin, F-actin, and IscU-like. These genes reside within the TGF-β/BMP, insulin/IGF-1, Wnt, JNK/p38, oxidative phosphorylation, UPRmt, Fe-S cluster assembly, and actin-dynamics regulatory networks, respectively. These pathways play key roles in regulating fundamental biological processes, including growth, development, lifespan, and stress responses.

### 3.8. Differential Gene Expression Analysis Between CK and S-Bx.Q2

**Figure 8:**
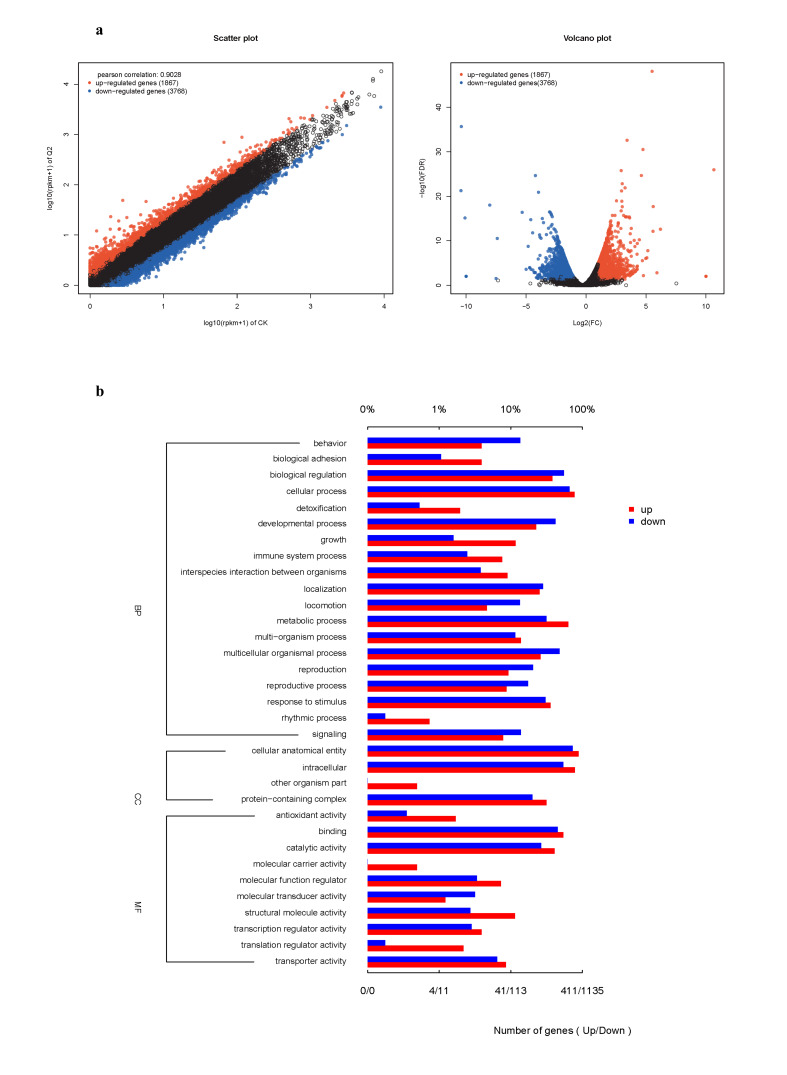
Functional annotation and enrichment analysis of DEGs between CK and S-Bx.Q2. (**a**) EDGs visualized by Scatter Plot and Volcano Plot. (**b**) Gene Ontology (GO) analysis of the upregulated and downregulated genes.

Comparative transcriptomic analysis between CK and S-Bx.Q2 identified 5465 DEGs (2122 upregulated, 3343 downregulated) (Figure 8a). GO enrichment revealed that in Biological Process, upregulated DEGs were enriched in cellular (320) and metabolic (263) processes, while downregulated DEGs dominated cellular (754) and regulatory (627) functions. In Cellular Component, both up- and downregulated DEGs primarily localized to cellular anatomical entities (363 upregulated, 831 downregulated) and intracellular regions (322 upregulated, 615 downregulated). In Molecular Function, binding (223 upregulated, 514 downregulated) and catalytic activity (169 upregulated, 301 downregulated) were most affected. Overall, downregulation was more prevalent, particularly in developmental processes (477) and transporter activity (73). Upregulated genes showed stronger involvement in stimulus response (147) and structural roles (47) (Figure 8b).

**Figure 9:**
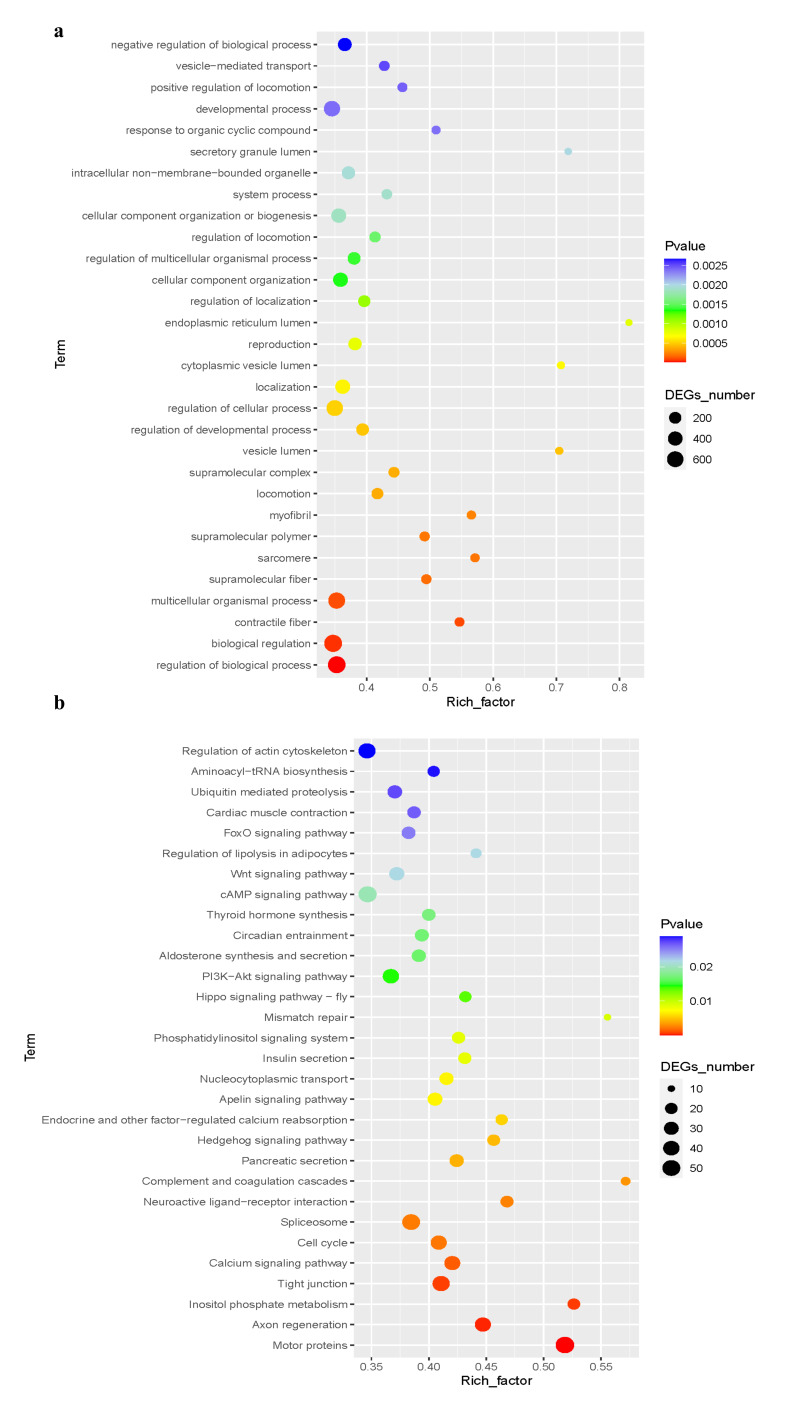
Functional annotation and enrichment analysis of DEGs between CK and S-Bx.Q2. (**a**) Scatter Plots of GO enrichment analysis. (**b**) Scatter Plots of enriched Kyoto Encyclopedia of Genes and Genomes (KEGG) pathways.

GO enrichment analysis of S-Bx.Q2 vs. CK revealed key functional categories dominated by downregulation: regulation of biological processes (745 DEGs: 144 upregulated, 601 downregulated), multicellular organismal processes (652:107 upregulated, 545 downregulated), developmental processes (570:93 upregulated,477 downregulated) and cellular regulation (607:128 upregulated, 479 downregulated). Notably, negative regulation (314 DEGs) and cellular component biogenesis (430 DEGs) also showed strong downregulation trends (Figure 9a).

KEGG enrichment analysis of DEGs between treatment S-Bx.Q2 and control CK revealed significant enrichment in the top 30 metabolic pathways. The most significant pathway, “Regulation of actin cytoskeleton,” suggested treatment impacted cell morphology and motility. Pathway enrichment—including “Aminoacyl-tRNA biosynthesis” and “Ubiquitin-mediated proteolysis”—indicated altered protein synthesis and degradation, likely reflecting cellular adaptation. Signal transduction pathways (“Cardiac muscle contraction,” “FoxO,” “cAMP,” and “Thyroid hormone synthesis”) highlighted complex regulatory effects on cellular physiology, emphasizing the roles of signaling molecules and hormones. Enrichment of “Mismatch repair” and “Phosphatidylinositol signaling system” further implied effects on DNA repair mechanisms and intracellular signaling, potentially influencing long-term cellular adaptability or damage responses (Figure 9b).

Transcriptomic differential expression analysis revealed significant downregulation of several key genes. As shown in Table 8, these included spondin-1, 14-3-3 zeta, daf-16-1 protein 2, SIR-2.1, Zona pellucida 2-like, cystatin-like, and vitellogenin-1. These genes are involved in neurogenesis/axon guidance, insulin/IGF-1 signaling, stress resistance, longevity control, energy metabolism, FOXO signaling, protease regulation, apoptosis, lipid metabolism, and reproduction. These pathways play key roles in neuronal development and maintenance, lifespan regulation, stress response, metabolic homeostasis, reproductive fitness, and cellular survival.

### 3.9. Validation of RNA-Seq Expression Data by RT-qPCR

**Figure 10:**
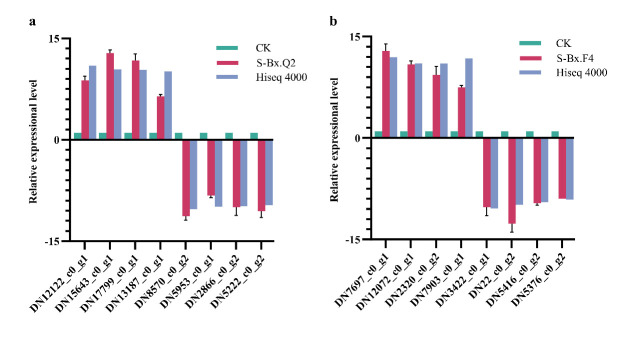
Transcriptome analysis validation of 16 differentially expressed genes (EDGs). (**a**) The comparison of CK and S-Bx.Q2; (**b**) The comparison of CK and S-Bx.F4..

To validate the differential expression identified by RNA-Seq, we selected 16 genes for RT-qPCR analysis. The primers used for validating the differentially expressed genes (DEGs) are listed in Table 2. The results showed no significant differences between the two assays, with error bars indicating standard deviation. A strong concordance was observed between the two methods, as 8 differentially expressed genes (DEGs) exhibited consistent expression trends, thereby confirming the reliability of our transcriptomic data. (Figure 10).

## 4. Discussion

*Bursaphelenchus xylophilus*, as a plant-parasitic nematode, harbors a diverse bacterial community on its surface, which is actively involved in its growth, reproduction, environmental adaptation, and infection of host pine trees [35]. The composition of these associated bacterial communities exhibits significant geographical heterogeneity [36]. Notably, significant differences in pathogenicity exist among different *B. xylophilus* isolates, which may be partly attributable to variations in the structure and function of their associated bacterial communities. Comparative studies of the bacterial communities associated with high- and low-virulence isolates have revealed that highly virulent nematodes often possess higher bacterial diversity and demonstrate enhanced capabilities in utilizing carbohydrates and carboxylic acids [14,37]. The presence of specific bacterial groups, such as *Stenotrophomonas* and *Pseudomonas*, is closely correlated with nematode virulence [37]. These findings indicate that the associated bacterial community serves as a critical regulator that shapes the pathogenicity of *B. xylophilus* through modulating its key physiological functions.

Certain dominant associated bacteria enhance the physiological activity and pathogenicity of *B. xylophilus* by providing nutritional support, improving environmental adaptation, or directly assisting in host invasion. Key to disease progression is the nematode’s reproductive capacity and its migration rate within the xylem, both of which jointly determine population expansion in the host and the development of pine wilt disease. Studies have shown that when co-cultured with *B. xylophilus*, *P. fluorescens* and *P. putida* can significantly increase egg production and reproduction rates, while obviously enhancing the nematode’s motility, specifically manifested as increased frequency of head swinging and body bending [38].

Furthermore, pine trees produce reactive oxygen species (ROS) as a defense response upon infection, and various associated bacteria can assist the nematode in coping with such oxidative stress. *Serratia* spp. possesses intrinsic antioxidant capacity, which not only significantly improves the survival of *B. xylophilus* under oxidative stress, but also upregulates the expression of nematode antioxidant enzyme genes (e.g., Bxy-ctl-1 and Bxy-ctl-2), thereby enhancing its ability to counteract host defenses and promoting successful colonization and survival during early infection [39]. On the other hand, certain associated or environmental bacteria can suppress the survival and reproduction of pine wood nematodes by producing inhibitory metabolites, highlighting their promise as potential biocontrol agents [40,41,42].

This study further revealed that the dominant associated bacteria isolated from highly pathogenic PWN significantly enhanced their motility, egg-laying capacity, population growth, lifespan, feeding ability, and pathogenicity. In contrast, when the dominant bacteria isolated from low-pathogenic PWN were introduced into highly pathogenic PWN, these parameters were markedly suppressed. These findings suggest that dominant associated bacteria may play a crucial regulatory role in the physiology and pathogenicity of PWN, providing a new theoretical foundation for the prevention and control of PWD. We propose the following hypothesis: in PWD, bacteria may function as conditional pathogens. Within the microbial community of healthy pine trees, these bacteria are generally harmless or even beneficial to the host. However, when they co-infect alongside pine wood nematodes, the bacteria may modulate the adaptability of the nematodes to the host by secreting specific metabolites or expressing their own detoxification-related genes, thereby influencing the occurrence and progression of PWD.

Our results showed that RNA sequencing revealed a significant impact on the gene expression of *B. xylophilus* treated with *A. radiobacter* Bx.F4 and *D. tsuruhatensis* Bx.Q2. according to RNA sequencing results, the analysis of differentially expressed genes between the S-Bx.F4 treatment group and the aseptic PWNs (CK) group (Table 7) revealed significant upregulation of several genes. Among them, the expression of MKK 7 was increased. As an upstream regulatory hub of the JNK signaling pathway, elevated MKK7 expression can enhance nematode environmental adaptability, homeostasis maintenance, and lifespan [43,44,45,46], providing a possible molecular explanation for the improved survival rate of nematodes treated with *A. radiobacter* Bx.F4.

Simultaneously, the expression of Axin, a core negative regulator of the Wnt/β-catenin signaling pathway, was also significantly increased. This pathway is involved in regulating embryonic development, cell fate determination, and organ formation, which may help explain the increased egg production and population growth observed after *D. tsuruhatensis* Bx.F4 treatment [47,48]. Furthermore, the expression of F-actin, a core cytoskeletal protein, was elevated. Its dynamic reorganization directly affects body wall muscle contraction patterns and pharyngeal pumping efficiency, potentially contributing to the enhanced locomotor ability and feeding range of the nematodes [49,50,51,52]. The expression of HSP60, a key molecular chaperone maintaining mitochondrial protein homeostasis, was also upregulated. By modulating pathways such as mitochondrial stress response, insulin/IGF-1 signaling, and apoptosis, HSP60 may further influence nematode development, locomotor ability, and lifespan [53,54,55].

In the comparison of differentially expressed genes between the S-Bx.Q2 treatment group and the aseptic PWNs (CK) group (Table 8), several genes showed significant downregulation. The expression of DAF-16, a core transcription factor in the DAF-16/FOXO signaling pathway and longevity-related pathways, was reduced by approximately 10.26-fold, while its cofactor SIR-2.1 was downregulated by 1.67-fold. This change may impair nematode lifespan and resistance to environmental stress and pathogens, potentially explaining the decreased survival rate and population growth after *D. tsuruhatensis* Bx.Q2 treatment [56,57,58,59]. Additionally, reduced expression of 14-3-3 zeta, a key adaptor in intracellular signaling, may further affect lifespan, stress resistance, and embryonic development, consistent with the observed decreases in survival rate, egg production, and population growth [60]. Moreover, decreased expression of cystatin-like protein, a cysteine protease inhibitor, may disrupt basic protein metabolism and growth development [61,62]. The downregulation of vitellogenin-1, a key yolk protein precursor [63], may impair nutrient transport from the intestine to the gonads, thereby compromising embryonic development and reproductive capacity. Together, these gene expression changes provide a molecular basis for the reduced survival rate, egg production, and population growth observed in nematodes treated with *D. tsuruhatensis* Bx.Q2.

In summary, the results revealed that their individual inoculation led to significant and even opposite trends in key physiological indicators of the nematodes. Transcriptomic analysis further demonstrated that the two bacterial strains differentially regulated the expression of multiple key signaling pathways and related functional genes in the nematodes. This indicates that their mechanisms of action are not limited to a single functional process; rather, they may systematically activate or inhibit interconnected gene expression networks in the nematodes through bacterial metabolites or other effector molecules, thereby enhancing or diminishing the overall health and fitness of the nematodes.

At the transcriptomic level, this study reveals that microbial symbionts can significantly influence host phenotypes by modulating the overall architecture of the host gene expression network, providing molecular evidence for the role of bacteria in the host adaptation of pine wood nematodes. To further elucidate the underlying mechanisms, follow-up studies could employ RNA interference technology for functional validation of key genes, combined with metabolomics analysis of metabolites produced by the associated bacteria. This approach would facilitate the screening of strains and metabolites with inhibitory activity for development as potential biocontrol agents. Subsequent field evaluations of their efficacy against pine wilt disease could offer a practical strategy for green control. Through this framework, the study aims to systematically clarify how these bacteria regulate host gene expression and their potential roles in the onset and progression of PWD.

## Figures and Tables

**Table 1:** Disease grade of pine wilt disease in *P. thunbergii*.

Disease Grade	Grading Standard	Representative Value
I	Healthy, normal growth of plants	0
II	A few needles turned yellow	1
III	Half of the needles turned yellow and the branches were bent	2
IV	Most of the needles turned yellow, tree became wilted	3
V	All the needles turned yellow, tree wilted	4

**Table 2:** The RT–qPCR primers for 16 differentially expressed genes.

ID	Forward Primer (5 → 3′)	Reverse Primer (5′ → 3′)
DN7697_c0_g1	GACGCTACTCAAGGACAAACAG	GGCTTCACTGTATATGGAGCTG
DN12072_c0_g1	TTCTCTTCCCTGTCATCTGCAG	AAGACCTCAGAAGCACTGGATG
DN2320_c0_g2	CAATCACTTACCCTGCTCACATG	TTTATAGCCGGGGATGCCATG
DN7903_c0_g1	CATCCTGAGTTCAAGTCTTGGC	GTTTTTGGCCACTAGACTGCTG
DN3422_c0_g1	TAGATTTCACGCTGCTAGAGACG	CAGGAAAATCTCGGTTCCAAGC
DN12122_c0_g1	GCGCATAATTGGAATTGCTCTC	GCAGTTGTAAGTGGTCCGATTC
DN5416_c0_g2	GTTTGCGGTCGACTATTTGCTC	CGAACCGCTCAAATACCAAGTC
DN5376_c0_g2	AAGAAGAAGGGGCATAGTGAGC	CGGTAACTGAATGAGGGAGTTG
DN12122_c0_g1	GCGCATAATTGGAATTGCTCTC	GCAGTTGTAAGTGGTCCGATTC
DN15643_c0_g1	GGGTAAAATCCGTTGATCCACTG	TCACCGAGTCTGCATCGTTTAC
DN17799_c0_g1	TTTGTCTGAACGGTCCTGCTG	CTGGAAATCCTTAGCTTCTTCAGG
DN13187_c0_g1	GATACGCAATGTGATGGCTGAC	CGTTGGCGATGACTTTCTGC
DN8570_c0_g2	TCCACAGCCAAGATACCAGAAAG	GTATTTTTCTGCCGCCTGTAACC
DN5953_c0_g1	GACGATTCCACTCTATGCGTTG	CGGAGCCTACAGAAAAGTTGATG
DN2866_c0_g2	CTACTAGAATCGACCCTTGAGACC	TAGCAAGAAGAGCTCGATGACTC
DN5222_c0_g2	TGGATGACGTCTACATTGTGGTC	CACAAGTGTAACAACCGGCTTC
Actin	GCAACACGGAGTTCGTTGTA	GTATCGTCACCAACTGGGAT

**Table 3:** Disease incidence and disease severity index (DSI) of *P. thunbergii* inoculated with differently treated pine wood nematodes (PWNs).

Treatments	Disease Incidence (%)		DSI	
	0 d	60 d	0 d	60 d
Sterilized water	0	0	0	0
S-Bx.F4	0	100	0	100
Aseptic PWNs	0	50	0	12.5
S-Bx.Q2	0	100	0	50

**Table 4:** Transcriptome data filtering statistics.

Category	Number of Reads	N50 (bp)	Total Bases	Mean Length of Unigenes (bp)
Raw data	398,182,724	-	-	-
Clean data	387,979,812	1818	23,322	1283

**Table 5:** Statistical summary of transcriptome quality control (QC) data.

Sample	Raw Bases (Gb)	Valid Bases (Gb)	Q20 (%)	Q30 (%)	GC (%)
CK-1	6.22	6.00	98.78	96.62	45.68
CK-2	6.37	6.16	98.95	97.09	46.15
CK-3	7.60	7.83	98.91	96.98	46.28
S-Bx.F4-1	6.72	6.52	98.88	96.88	46.19
S-Bx.F4-2	6.83	6.20	98.95	97.09	46.21
S-Bx.F4-3	6.86	6.67	98.91	96.99	46.20
S-Bx.Q2-1	6.24	6.03	98.87	96.88	45.92
S-Bx.Q2-2	6.30	6.10	98.75	96.53	45.75
S-Bx.Q2-3	7.03	6.82	98.90	96.96	45.00

**Table 6:** Summary of annotation of Unigenes in each database.

Public Protein Database	Number of Unigenes	Annoted Unigenes	Percentage (%)
NR	23,322	17,833	76.5
GO	23,322	5014	21.5
COG	23,322	11,027	47.3
KEGG	23,322	6052	25.9
SWSS	23,322	9116	39.1

**Table 7:** Upregulated EDGs in sample S-Bx.F4 compared to CK.

Genes ID	Description	Fold Change	q-Value
DN16175_c0_g1	Astacin protease inhibitor	10.53	1.32 × 10^−5^
DN11712_c0_g1	Cytochrome c oxidase subunit II	1.77	0.001597
DN633_c0_g1	Ribosyldihydronicotinamide dehydrogenase	4.15	7.16 × 10^−5^
DN5969_c0_g1	Zona pellucida 2-like	4.00	0.000206
DN8936_c0_g1	Zona pellucida 4-like	3.55	4.28 × 10^−13^
DN8003_c0_g1	Extracellular superoxide dismutase	11.23	7.16 × 10^−5^
DN19317_c0_g1	Zona pellucida 3-like	10.95	2.53 × 10^−5^
DN8807_c0_g1	MKK 7	2.03	3.57 × 10^−8^
DN7368_c0_g1	Axin	3.08	3.38 × 10^−9^
DN18448_c0_g1	IscU-like	9.81	4.27 × 10^−6^
DN71438_c0_g1	F-actin	2.08	2.04 × 10^−12^

**Table 8:** Downregulated EDGs in sample S-Bx.Q2 compared to CK.

Genes ID	Description	Fold Change	q-Value
DN8570_c0_g2	spondin-1-like	10.26	0.0517
DN8936_c0_g3	14-3-3 zeta	2.55	0.0087
DN8700_c0_g5	daf-16-1 protein 2	10.9	1.66 × 10^−7^
DN3518_c0_g2	SIR-2.1	1.67	1.22 × 10^−7^
DN8936_c0_g3	Zona pellucida 2-like	9.32	8.23 × 10^−7^
DN8854_c0_g1	cystatin-like	9.84	0.0052
DN16302_c0_g1	Vitellogenin-1	2.94	0.0032

## Data Availability

The original contributions presented in this study are included in the article and Supplementary Materials. Further inquiries can be directed to the corresponding authors.

## Supplementary Materials

The following supporting information can be downloaded at https://www.mdpi.com/article/10.3390/microorganisms14010010/s1, Figure S1: Microscopic observation of PWN stained with crystal violet after sterilization treatment (×1000). A: Sterilized PWN stained with crystal violet. B: Wild PWN stained with crystal violet. Figure S2: PCR amplification of 16S rRNA gene from bacteria associated with PWN. 1–4: Aseptic *B. xylophilus*. 5: 2K plus DNA marker. 6: Wild *B. xylophilus* isolate 1. 7: Wild *B. xylophilus* isolate 2.
