# Supplementary material for: Effects of Dominant Associated Bacteria Agrobacterium radiobacter Bx.F4 and Delftia tsuruhatensis Bx.Q2 on the Physiological Traits of Bursaphelenchus xylophilus: Insights from RNA-Seq Analysis
Source: Microorganisms. 2025 Dec 19;14(1):10. doi: 10.3390/microorganisms14010010 (PMC12844259; doi:10.3390/microorganisms14010010)
Supplement: Supplementary file 1 [file microorganisms-14-00010-s001.zip › microorganisms-3997396-supplementary.pdf]

Supplementary Materials

Figure S1

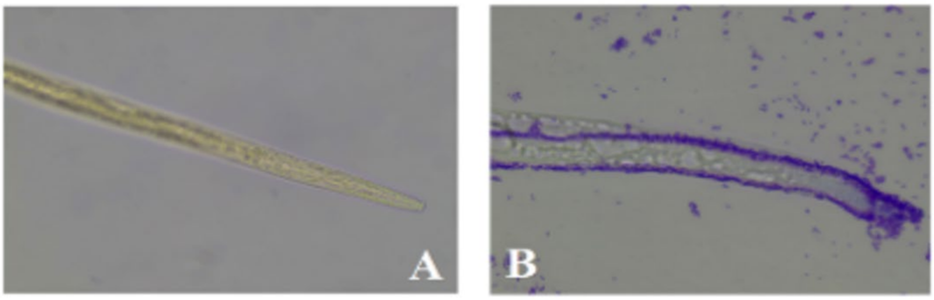

Figure S1. Microscopic observation of PWN stained with crystal violet after sterilization treatment（×1000）. A: Sterilized PWN stained with crystal violet. B: Wild PWN stained with crystal violet.

Figure S2

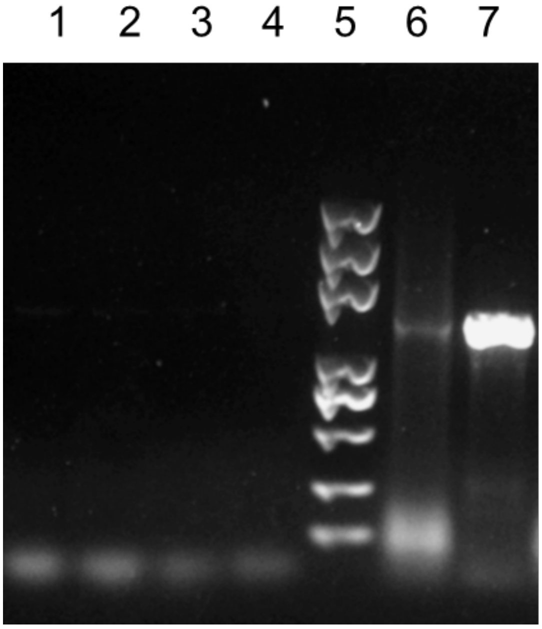

Figure S2. PCR amplification of 16S rRNA gene from bacteria associated with PWN. 1-4: Aseptic *B. xylophilus*. 5: 2K plus DNA marker. 6: Wild *B. xylophilus* isolate 1. 7: Wild *B. xylophilus* isolate 2.
